# Autonomic Dysfunctions in Parkinson's Disease: Prevalence, Clinical Characteristics, Potential Diagnostic Markers, and Treatment

**DOI:** 10.1155/2020/8740732

**Published:** 2020-12-24

**Authors:** Zhe Zhang, Sheng-Di Chen

**Affiliations:** ^1^Department of Neurology, Ruijin Hospital Affiliated to Shanghai Jiao Tong University School of Medicine, Shanghai, China; ^2^Department of Neurology, Peking Union Medical College Hospital, Beijing, China

## Abstract

Parkinson's disease (PD) is a common neurodegenerative disease in the middle-aged and the elderly. Symptoms of autonomic dysfunctions are frequently seen in PD patients, severely affecting the quality of life. This review summarizes the epidemiology, clinical manifestations, and treatment options of autonomic dysfunctions. The clinical significance of autonomic dysfunctions in PD early diagnosis and differential diagnosis is also discussed.

## 1. Introduction

Parkinson's disease (PD) is a common neurodegenerative disease in the elderly with the secondary highest prevalence to Alzheimer's disease [1]. Worldwide and in China, both the numbers of PD patients and the health burden increase rapidly [2]. In addition to the classical motor symptoms of tremor, rigidity, bradykinesia, and postural instability, more attention has been paid to nonmotor symptoms, including olfactory dysfunctions, sleep disorders, apathy, anxiety, depression, cognitive impairment, and autonomic dysfunctions; among them, autonomic dysfunctions are prominent symptoms of PD, which severely affects the quality of life of PD patients and causes a huge burden on the patients, their caregivers, and the health economic system [3, 4]. According to a longitudinal study assessing autonomic symptoms in PD patients at the Hoehn–Yahr stage 1 with disease duration <2 years and following up those patients for three years, at least one symptom of autonomic dysfunctions was present in 71% of the PD patients in early stages and in all patients after 3-year follow-up [5].

In this review, we will discuss the prevalence, clinical manifestations, and treatment of autonomic dysfunctions in PD, as well as the clinical significance of autonomic dysfunctions in early diagnosis and differential diagnosis of PD.

## 2. Prevalence of Autonomic Dysfunctions in PD

The common cardiovascular autonomic dysfunctions in PD are orthostatic hypotension (OH) and its related symptoms. Senard et al. first carried out the epidemiological study of OH in PD in 1997 [6]. The researchers measured the blood pressure of 91 PD patients who stood for 10 minutes after lying supinely for 15 minutes. The related symptoms, including dizziness, postural instability, vertigo, blurred vision, syncope, fatigue, and hearing loss, were also documented. The prevalence of OH in PD patients was 58.2% (53/91), of which 19.8% (18/91) had corresponding symptoms. Jost and Augustis measured the blood pressure before and after the postural change of 106 PD patients, finding that the severity of OH, defined as maximal drop of systolic blood pressure after postural challenge, was not correlated with the course of disease [7]. Similar studies have shown that the prevalence of OH in PD is 47% and that the prevalence of OH does not vary with the course and severity of the disease [8]. A meta-analysis study, which included 25 studies and 5070 PD patients, estimated that the prevalence of OH in PD was 30.1% [9].

Gastrointestinal dysfunctions of PD can be manifested as weight loss, sialorrhea, dysphagia, symptoms related to gastric emptying disorder, constipation, and so on. Abbott et al.'s study showed that weight loss was observed in 52% of PD patients, and more than 22% of PD patients lost more than 28 pounds [10]. The results of the latest epidemiological survey in 2018 were similar to the study above, with 48.6% of PD patients suffering from weight loss compared to 20.8% of healthy controls [11]. In an epidemiological survey of 420 PD patients, the researchers used Scales for Outcomes in Parkinson's Disease-Autonomic Dysfunction (SCOPA-AUT) to assess the prevalence of autonomic dysfunctions in PD patients [12]. The study showed that 73.1% (307/420) of PD patients had sialorrhea and the proportion increased to 88% (370/420) as disease progressed. In the newly diagnosed and untreated PD patients, the incidence of sialorrhea could be as high as 42% [13]. About 30% to 82% of PD patients reported dysphagia subjectively [14], and modified barium swallowing study (MBSS) showed abnormality in 75% to 97% PD patients [15]. Goetze et al. studied the prevalence of gastric emptying disorder in PD patients by feeding 80 patients with foods labeled with different isotopes [16]. The researchers found that the prevalence of solid food emptying disorder and liquid food emptying disorder in PD patients was 87.50% (35/40) and 37.50% (15/40), respectively. Small intestine bacteria overgrowth (SIBO) was also common in PD patients, reflecting the underlying intestinal hypomotility in PD [17]. The prevalence of SIBO in PD patients was 30.2% compared to 9.5% in healthy controls. Constipation is another manifestation of gastrointestinal hypomotility. The prevalence of constipation in patients with PD varies with different research methods. Through SCOPA-AUT survey, the subjective complaint of “constipation” was 80% in PD patients [12]. Another pilot study showed that 59% of PD patients and 20.9% of healthy controls were diagnosed as constipation in accordance with the Rome criteria, and 38.4% of PD patient were treated with laxatives, compared to 14.2% in healthy controls [18].

About 27% to 39% PD patients had symptoms of urinary system dysfunctions, which could be categorized into irritative and obstructive symptoms [19]. The incidence of detrusor hyperreflexia was as high as 45 to 100%, while the incidence of obstructive symptoms (such as thinning of urine flow, difficulty in urination, and bladder emptying disorders) was 27% [14].

The prevalence of sexual dysfunctions was high in both male and female PD patients. The prevalence of erectile dysfunction, premature ejaculation, ejaculation difficulty, and decreased libido in male PD patients ranged from 44% to 79%, the prevalence of decreased libido in female PD patients ranged from 46.9% to 70%, and the prevalence of orgasm difficulty in female PD patients was 75.0% [20–24].

## 3. Clinical Manifestations of Autonomic Dysfunctions in PD

**Figure 1:**
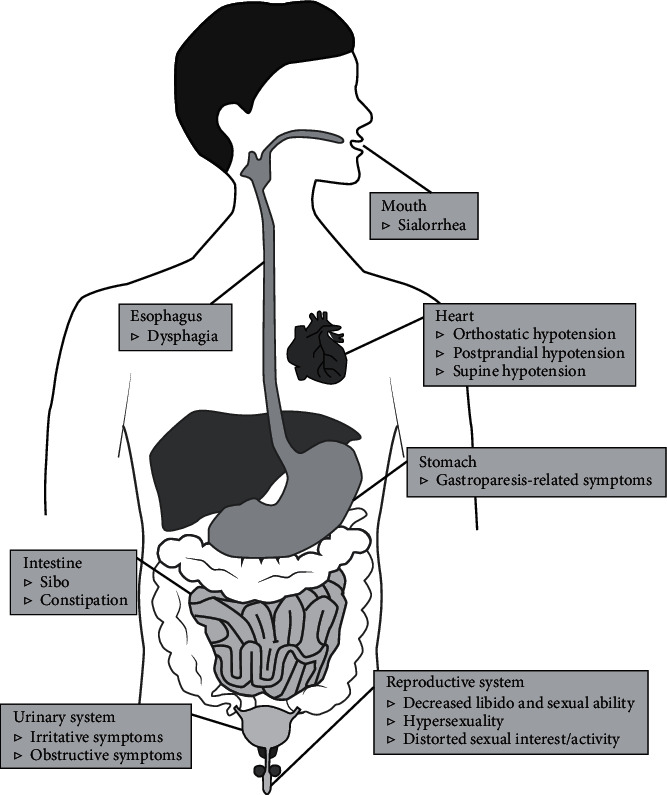
Clinical manifestations of autonomic dysfunctions in PD. SIBO: small intestine bacterial overgrowth.

Autonomic dysfunctions in PD can involve the sympathetic noradrenergic system (SNS), the sympathetic cholinergic system (SCS), the sympathetic adrenomedullary system (SAS), the parasympathetic nervous system (PNS), and the enteric nervous system (ENS) manifested as symptoms and signs of cardiovascular system, digestive system, urinary system, reproductive system and skin, and other systems (Figure 1).

### 3.1. Autonomic Dysfunctions of Cardiovascular System

The cardiovascular system is mainly dominated by SNS, SAS, and PNS. The main manifestations of cardiovascular autonomic dysfunctions are orthostatic hypotension and postprandial hypotension with or without the clinical symptoms of dizziness, blurred vision, “vague” thinking, fatigue, nape pain, and so on. OH, according to the consensus statement, is defined currently as sustained drop of systolic blood pressure of at least 20 mm·Hg or diastolic blood pressure of at least 10 mm·Hg within 3 minutes upon sitting or head-up tilting of at least 60 degrees [25]. OH in PD is considered to be neurogenic, resulting from degeneration of postganglionic efferent sympathetic neurons in baroreflex cycle [26]. Therefore, OH in PD patients should be carefully differentiated from nonneurogenic OH caused by decreased pumping capacity of the heart and intravascular volume loss or redistribution. Common conditions include heart failure, dehydration, and medications (such as *β* blockers, diuretics, and calcium channel blockers). One practical method to distinguish neurogenic versus nonneurogenic OH is the ratio of heart ratio changes to systolic blood pressure changes (ΔHR/ΔSBP). A ΔHR/ΔSBP less than 0.492 bpm/mm·Hg in neurogenic OH had high sensitivity (91.3%) and specificity (88.4%) to be differentiated from nonneurogenic OH [27]. It is worth mentioning the side effects caused by the usage of levodopa, which may induce vasodilation and diuresis, and therefore aggravate the symptoms related to OH. However, hypotension caused by levodopa is not postural. Studies have shown that the occurrence of OH in PD is not related to levodopa usage [28]. Orthostatic hypotension has been identified as a modifiable risk factor for cognitive impairment in PD [29]. Additionally, OH in PD has been shown to be associated with postural instability, incidental falls, and decreased survivals, suggestive of more active intervention [30]. Postprandial hypotension is usually triggered by a high-carbohydrate food, which may last about 3 hours starting after 15 minutes after eating, possibly due to splanchnic vasodilation and intravascular volume redistribution. Abnormal blood pressure regulation in PD can also manifest as supine hypertension. Supine hypertension is defined as systolic blood pressure more than 140 mm Hg and/or diastolic blood pressure more than 90 mm·Hg when measured after 5-minute supine resting [31]. There is a high incidence of supine hypertension not only in PD patients with OH, but also in PD patients without OH, with prevalence of 95% and 79%, respectively [32]. In addition, supine hypertension in PD patients increases the frequency of target organ damage and is a risk factor for stroke and cardiovascular events [33]. Additionally, the “off-period” of PD is related to hypertension, higher resting heart rate, and higher change of blood pressure during postural changes [3]. In addition to dysregulation of blood pressure, cardiovascular autonomic dysfunctions in PD can also be characterized by chronotropic insufficiency, that is, insufficient increase of heart rate in response to stress, such as exercise [34, 35]. Chronic insufficiency may partially explain the symptoms of fatigue and exercise intolerance in PD patients.

### 3.2. Autonomic Dysfunctions of Gastrointestinal System

Weight loss, sialorrhea, dysphagia, gastroparesis-related symptoms, intestinal dyskinesia-related symptoms, and constipation are the clinical manifestations of gastrointestinal autonomic dysfunctions in PD patients, which are discussed below.

PD patients had a stable body weight 2 to 4 years before diagnosis but continued to decline in body weight after diagnosis. PD patients lost about 5.2 pounds in average in 10 years before diagnosis and 7.7 pounds in eight years after diagnosis [36]. Poor nutritional status is associated with weight loss in PD patients. A pilot study showed that the prevalence of malnutrition in PD patients was 1.71%, and 19.66% of PD patients were at risk of malnutrition. Poor nutritional status was associated with constipation, vomiting, and emotional disorders [37].

Sialorrhea in PD patients is not due to increased salivary gland secretion. In fact, studies have shown a decrease in salivary gland secretion in PD patients [38]. Multiple factors contribute to drooling in PD, including decreased swallowing capacity, dysphagia, unintentional mouth opening due to hypomimia, and stooped posture, with hypomimia the most prominent one [39].

Dysphagia in PD patients can occur in all phases of swallowing, including oral phase, pharyngeal phase, and esophageal phase. Rigidity and bradykinesia of striated muscles involved in oral and pharyngeal phases, dyskinesia of esophageal smooth muscle and striated muscles, and sensory impairment of pharynx, are all involved in the occurrence of dysphagia in PD patients. Attention should be paid to differentiate with other diseases that cause dysphagia, such as achalasia, gastroesophageal reflux, esophageal diverticulum, and so on. PD patients may be complicated with these diseases. The treatment for these diseases may be completely different. The risk associated with dysphagia is aspiration. Modified barium swallowing study (MBSS) showed that about 46% of PD patients had aspiration with a higher prevalence in solid food aspiration, although these aspiration events did not always cause clinical symptoms [40].

Symptoms related to gastroparesis include loss of appetite, early satiety, nausea, vomiting, abdominal distension, and weight loss. Gastroparesis-related symptoms may occur secondary to PD or are adverse reactions of anti-Parkinsonian drugs, which requires to be carefully clarified for rational drug usage. Wireless motility capsule and gastric scintigraphy are sensitive diagnostic tools for gastroparesis [41]. Gastroparesis can also cause pharmacokinetic changes of levodopa with clinical manifestations as less responsiveness to levodopa or even complete failure of levodopa.

Intestinal dyskinesia in PD may lead to small intestine bacteria overgrowth (SIBO). However, SIBO is not merely the consequence of PD but may contribute to PD pathogenesis by promoting neuroinflammation and migration of alpha-synuclein aggregates along the gut-brain axis [42]. SIBO also leads to worse motor symptoms in PD clinically [43]. The interplay between changes of gut microbiome and pathogenesis of PD is complex and discussed in detail in a recent review [44]. SIBO can cause abdominal distension and less responsiveness to levodopa [43]. Lactulose breath test may be helpful for diagnosis [45]. Gut hypomotility due to degeneration of myenteric dopaminergic neurons plays key roles in pathogenesis of constipation. Additionally, decreased physical activity and medications, including anticholinergic drugs and dopaminergic agonists, may also contribute to constipation in PD patients [46]. In addition to symptomatic diagnosis, objective tools including colonic transit time evaluated by the radio opaque marker technique, computed tomography based volume estimation, and gastric emptying time by scintigraphy, are all very sensitive for constipation diagnosis [47]. In addition to constipation, defecation disorders in patients with PD can also show exertional defecation, painful defecation, and incomplete defecation.

### 3.3. Autonomic Dysfunctions of Urinary System

The autonomic dysfunctions of urinary system can be manifested as irritative symptoms and obstructive symptoms. The most common irritative symptoms in PD patients include nocturia, followed by urinary urgency and frequency. Acute urinary incontinence is frequently seen in PD patients with obvious dyskinesia [48, 49]. The obstructive symptoms of urinary system in PD include urinary hesitation, urinary exertion, thinning of urine flow, and incomplete emptying. These symptoms need to be carefully differentiated from prostate hyperplasia, which is a common comorbidity in elderly PD patients. PD itself can also cause obstructive symptoms mainly due to decreased activity and sensation of detrusor, as well as delayed relaxation of urethral sphincter [19].

### 3.4. Autonomic Dysfunctions of Reproductive System

Autonomic dysfunctions of reproductive system in PD patients included decreased libido and sexual ability, hypersexuality, and distorted sexual interest/activity. Hypersexuality and distorted sexuality may be caused by hedonistic homeostatic dysregulation, which is related to improper use of levodopa and dopaminergic receptor agonists [50]. These symptoms include increased libido, sexual addiction, obsessive-compulsive sexuality, and impulsive sexual behavior, such as purchase of pornographic services and exhibitionism [20]. The more common sexual dysfunctions in PD patients are decreased libido and decreased sexual ability. In male PD patients, low libido, erectile dysfunction, premature ejaculation, difficulty in ejaculation, and low self-esteem are frequently seen. Female PD patients may manifest as reduced libido, decreased vaginal sensitivity, vaginal spasm, difficulty in reaching orgasm, and low self-esteem [51]. A recent prospective longitudinal study which included 355 men with early PD, namely, H-Y stage ≤2, showed that higher sexual activity was associated with slighter motor disability, milder depression, and better quality of life, promoting that more attention should be paid to identify and treat sexual dysfunctions in early PD patients [52].

### 3.5. Autonomic Dysfunctions of Other Systems

Autonomic dysfunctions of PD can also be manifested as thermoregulatory dysfunctions, such as high sensitivity to temperature, abnormal sweating, and abnormal body temperature. Temperature sensitivity includes cold intolerance and hot intolerance with the former one more common [53]. Abnormal sweating can be manifested as decreased sweating of the trunk, increased sweating of head, face, and limbs, increased sweating of the affected side, and intermittent sweating [54, 55]. It has been shown recently that chronic hyperhidrosis was associated with higher rate of nonmotor symptoms, such as sleep disorders, anxiety, depression, dyskinesia (fluctuation-related hyperhidrosis), and worse quality of life [56]. Abnormal body temperature in PD patients is typically paroxysmal high fever, especially in sudden withdrawal of anti-Parkinson's drugs and in “off-phase” [57, 58].

## 4. Treatment Options for Autonomic Dysfunctions in PD

In this part, we briefly introduce the treatment options for autonomic dysfunctions in PD patients system by system corresponding to the clinical manifestations we discussed above.

Management of OH in PD emphasizes on reduction of symptom burden instead of strictly normalizing blood pressure. Symptoms of OH should routinely be asked in clinical practice to identify those OH patients with symptoms. Postprandial hypotension and OH with no or mild clinical symptoms can be treated by behavior guidance to slowly change position and to eat low-carbohydrate food. While for PD patients with severe symptomatic OH, pharmacological and nonpharmacological interventions should be applied in combination to achieve better symptom relief. Nonpharmacological interventions include correction of aggravating factors, such as those mentioned above in nonneurogenic OH, and behavior guidance includes increased water and salt intake, lying with head elevated, wearing waist-high tight stockings, and recumbent exercises [26]. Medications include drugs restoring vascular tone, such as midodrine, and drugs that increase water and salt retention, such as fludrocortisone [14]. Attention should be paid to avoid aggravating supine hypertension when correcting OH. Ambulatory blood pressure monitor may be applied to achieve a better balance between OH and supine hypertension. The treatment of OH in PD and achievement of a compromise between OH and supine hypertension is discussed in detail in a recent review by Palma and Kaufmann [26].

For PD patients with gastrointestinal dysfunctions, weight loss, and malnutrition in particular, regular nutrition assessment is necessary. In addition, optimizing drugs for motor symptoms and nonmotor symptoms is helpful for the treatment of malnutrition in PD. Before levodopa treatment, patients are advised to take balanced Mediterranean-like dietary regimen while a low-protein diet is recommended at the advanced stage of the disease [59]. For patients with mild sialorrhea, behavior treatment strategies should be offered first. Patients can be instructed to suck hard candies in their mouths or to chew gum to increase the conscious swallowing. Severe sialorrhea can be treated with anticholinergic drugs such as glycopyrrolate that do not cross the blood-brain barrier or sublingual administration of atropine eye drops [60]. Dihydroergotoxine mesylate, which is an alpha-adrenergic blockers with affinities to dopaminergic and serotonin (5-HT) receptors, has been shown by recent studies to be safe and effective for sialorrhea in PD patients [61]. If the symptoms remain uncontrolled, botulinum toxin can be injected into the salivary glands [62–64]. The treatment of dysphagia depends on multidisciplinary teams. MBSS is necessary for assessing PD patients with dysphagia. If MBSS indicates that dysphagia occurs in the oropharynx and upper esophagus, rehabilitation therapy for swallowing function may be helpful. If MBSS suggests that dysphagia in PD patients is caused by lower esophageal dysfunction, esophagoscopy is recommended. If MBSS suggests that dysphagia in PD patients is caused by other diseases such as esophageal diverticulum, referrals to relevant specialists are recommended [15]. The efficacy of levodopa in treatment of dysphagia has yet to be confirmed [65, 66]. For patients with gastroparesis-related symptoms, metoclopramide should not be used because metoclopramide can pass the blood-brain barrier and antagonize dopamine receptors in the central nervous system. Peripheral dopamine receptor antagonists such as domperidone can be used to promote gastric motility and improve gastroparesis. Antibiotic treatment of SIBO might improve the drug response of PD patients to levodopa [67, 68]. Constipation in PD patients should be treated in a stepwise manner with the first-line therapy to increase dietary fiber and liquid intake, such as supplementation of psyllium and methylcellulose [69]. If the above treatment is ineffective, laxatives can be selected. Irritant laxatives such as bisacodyl and sennosides are not suitable, but osmotic laxatives such as lactulose and polyethylene glycol (PEG) are often effective [70]. Third-line therapy including lubiprostone and linaclotide. Patients with defecation disorders can be treated by rehabilitation therapy such as pelvic muscle exercise [71]. In addition, subcutaneous injection of morphine and local injection of botulinum toxin into the external anal sphincter and pubic rectum muscle are effective [72, 73].

The medication for irritative urinary symptoms mainly includes cholinergic receptor antagonists to combat bladder hyperreflexia, including oxybutynin and tolterodine. M3 receptor blockers, including darifenacin, solifenacin, and trospium, have fewer adverse reactions because of their selectivity to bladder and of not passing through the blood-brain barrier. In addition, local injection of botulinum toxin into detrusor under cystoscope and percutaneous posterior tibial nerve stimulation is also an option [74, 75]. A randomized controlled study of effects of tibial nerve stimulation on urinary problems in PD patients which are in progress deserves attention [76]. Bladder training was also found to significantly reduce the number of voids in 24 hours and improve the quality of life [77]. For nocturia, extended release of levodopa administered at bed-time was also found to be effective for PD patients [78]. Additionally, based on altered brain-bladder relationship in PD patients, recent several independent studies found that deep brain stimulations significantly alleviated urinary dysfunctions including urinary frequency, urgency, incontinence, and nocturia [79, 80]. Treatment of the obstructive urinary symptoms depends on the etiology. If obstructive symptoms are caused by prostate hyperplasia, choices include *α*-adrenergic receptor antagonists and 5-*α* reductase inhibitors, or surgery. If the symptoms are caused by detrusor inactivity, it is very important to reduce or stop the use of cholinergic receptor antagonists. And levodopa is used for delayed relaxation of urethral sphincter [81]. If the above measures do not work, symptomatic treatment including urethral catheterization should be considered [82].

In treatment of reduced libido and decreased sexual ability in PD patients, it is necessary to carefully identify other causes of the symptoms above, such as aging, hyperlipidemia with vascular problems, diabetes mellitus with neurovascular complications, mental disorders, and so on. Sildenafil is effective in treatment of erectile dysfunction. However, it should be noted that the use of sildenafil may cause OH in PD patients. Dopaminergic drugs such as levodopa and dopamine receptor agonists, working by stimulating D2 receptor in preoptic area, inhibiting prolactin release, and increasing serum oxytocin level, can be used to treat reduced libido and decreased sexual ability [83]. Ropinirole, levodopa, and apomorphine are also effective. It is worth noting that improper use of these drugs may result in hypersexuality and distorted sexuality.

For patients with thermoregulatory dysfunctions, the key of treatment is the rational use of anti-Parkinson's drugs, such as avoiding sudden anti-PD drug withdrawal and minimizing the period of off-phase.

## 5. Clinical Significance of Autonomic Dysfunctions in PD Early Diagnosis and Differential Diagnosis

The pathological study of 98 patients without mental and neurological diseases carried out by Bloch et al. showed that *α*-synucleinopathy was detected in 17 patients [84]. A further pathological study of these 17 idiopathic Lewy body disease (iLBD) patients showed that *α*-synucleinopathy was found in brainstem, olfactory nerve, thoracic nucleus of the spinal cord, sacral parasympathetic nucleus, esophageal myenteric plexus, and sympathetic ganglion. This study suggests that, in addition to medulla oblongata and olfactory system, autonomic nervous system is also the first site where pathological changes occur [85]. Another pathological study of abdominal and pelvic surgical specimens from 100 patients without neurological diseases showed that *α*-synucleinopathy was positive and distributed in autonomic plexus in 6 patients [86]. In the follow-up study of the six patients, it was found that the myocardial MIBG uptake was decreased and the recovery of blood pressure after Valsalva maneuver was slower in the six patients than in the control group. These two studies show that the pathological changes occur early in peripheral autonomic nervous system in iLBD, pathologically suggesting that autonomic dysfunctions may be a biomarker for early diagnosis of PD.

Goldstein et al. reported a case in which 18F-DOPA imaging showed a significant concentration decrease in left ventricular four years before onset of the motor symptoms in PD, suggesting that cardiac SAS denervation may occur in the early stage of PD [87]. Pathological studies have found that, in iLBD patients, cardiac sympathetic denervation occurs even earlier than the pathological changes of dorsal motor nucleus of vagus nerve in medulla oblongata [88]. It is suggested pathologically that cardiac sympathetic dysfunction can be used as a biomarker for early diagnosis of PD. In addition, the severity of cardiac sympathetic denervation was correlated with the disease course and the severity of PD, indicating that cardiovascular autonomic dysfunctions can also be used as a marker for progression of PD [89]. As mentioned earlier, cardiovascular autonomic dysfunctions in PD can be characterized by chronotropic insufficiency. A retrospective study has shown that chronotropic insufficiency is a biomarker for early diagnosis of PD. Under maximal exercising condition, the sensitivity and specificity of “maximal heart rate less than 143 bpm” were 83% and 62% in predicting PD after an average of 4.27 years. It is controversial that orthostatic hypotension is a biomarker for early diagnosis of PD. A retrospective study of 34 PD + OH patients showed that 58.82% (20/34) of patients reported OH before, concurrently with or within one year after the onset of PD motor symptoms [90]. Larger clinical studies have shown that OH is not a risk factor for PD, but this study suggests that abnormal electrocardiogram and carotid atherosclerosis are risk factors for PD [91].

Constipation is the most obvious early symptom of autonomic gastrointestinal system dysfunctions in PD patients. A pathological study has shown that *α*-synucleinopathy appeared in the colon of PD patients 2 to 5 years before motor symptoms onset [92]. Another pathological study showed that the prevalence of iLBD decreased with the increased frequency of defecation [93]. Both basic and clinical research has indicated that constipation is a potential biomarker for early PD. In human A53T *α*-synuclein transgenic mice, signs of gastrointestinal dysfunctions, demonstrated by delayed food transit along the GI tract, reduced electrically evoked motor response of the colon and presence of alpha-synucleinopathy in the ENS,preceded motor symptoms and CNS alpha-synucleinopathy by at least half a year [94]. The first clinical study to demonstrate that constipation was a risk factor for PD came from the Honolulu Heart Program, in which 6790 people without PD was followed up for a long period of time [95]. The researchers found that subjects with bowel movement less than once a day were 2.7 times more likely to develop PD than those who defecated once a day and 4.1 times more likely to develop PD than those who defecated twice a day. According to a case-control study [174], constipation in PD patients can occur more than 20 years before motor symptoms and is a risk factor for PD [96]. Perspective studies have led to similar conclusions. The Health Professionals Follow-Up Study showed that men with bowel movement less than once every three days were 4.98 times more likely to develop PD than men with daily defecation. The Nurses' Health study showed that women who defecated less than once every three days were 2.15 times more likely to develop PD than those who defecated daily [97].

A study of idiopathic rapid eye movement sleep behavior disorder (iRBD) patients suggests that erectile dysfunction is a risk factor for *α*-synucleinopathy diseases [98]. The sensitivity of erectile dysfunction in diagnosis of *α*-synucleinopathy diseases five years before disease onset was 63% to 71% with a specificity of 92%. Another study showed that in people aged 60 and over 60, 50 to 59, and younger than 50 with erectile dysfunction in comparison to those without erectile dysfunction, the relative risks of developing PD were 2.7, 3.7, and 4.0, respectively [99].

As one of the three major *α*-synucleinopathy diseases, PD needs to be differentiated from the other two *α*-synucleinopathy diseases, MSA, and dementia with Lewy Body (DLB), as all three diseases share similar pathology and autonomic dysfunctions. Pathologically, PD and DLB are characterized by intracytoplasmic *α*-synuclein inclusions and neurites in neurons while MSA are characterized by *α*-synuclein-positive glial cytoplasmic inclusions in the oligodendroglia [100]. Clinically, autonomic dysfunctions of PD often need to be differentiated from those of MSA and DLB, especially MSA-P. The sensitivity and specificity of myocardial 6–18F-DOPA PET imaging in differentiating PD from MSA were 83% and 80%, respectively [101]. Additionally, urodynamics and sphincter motor unit potential examination can help distinguish PD from MSA. All these following findings are highly suggestive of MSA, including the residual urine more than 100 mL, dyssynergia of detrusor and external urethral sphincter, bladder neck opening at the beginning of bladder filling, and the denervated potential of sphincter motor unit [102]. A recent urodynamic study consistent with the findings mentioned above further found that PD patients had higher incidence of detrusor overactivity and associated leakage and that residual volume from a pressure-flow study has the best sensitivity and specificity for differentiating PD from MSA [103].

## 6. Concluding Remarks

Autonomic dysfunctions, which involve the cardiovascular system, gastrointestinal system, urinary system, reproductive system, and so on, are frequent nonmotor symptoms in PD patients, severely affecting the quality of life. Because of relative less attention paid, autonomic dysfunctions are occasionally misdiagnosed and delayed for treatment. Additionally, autonomic dysfunctions are clinically useful for PD early diagnosis and differential diagnosis and are helpful for assessing disease progression and prognosis.
